# Multisite transformation in *Neisseria gonorrhoeae*: insights on transformations mechanisms and new genetic modification protocols

**DOI:** 10.3389/fmicb.2023.1178128

**Published:** 2023-06-20

**Authors:** Vui Yin Seow, Olga Tsygelnytska, Nicolas Biais

**Affiliations:** ^1^Brooklyn College of the City University of New York, Brooklyn, NY, United States; ^2^The Graduate Center of the City University of New York, New York, NY, United States; ^3^Laboratoire Jean Perrin, UMR8237, Sorbonne Université, Paris, France

**Keywords:** genome editing, Neisseria, natural transformation, co-transformation, DNA uptake

## Abstract

Natural transformation, or the uptake of naked DNA from the external milieu by bacteria, holds a unique place in the history of biology. This is both the beginning of the realization of the correct chemical nature of genes and the first technical step to the molecular biology revolution that sees us today able to modify genomes almost at will. Yet the mechanistic understanding of bacterial transformation still presents many blind spots and many bacterial systems lag behind power horse model systems like *Escherichia coli* in terms of ease of genetic modification. Using *Neisseria gonorrhoeae* as a model system and using transformation with multiple DNA molecules, we tackle in this paper both some aspects of the mechanistic nature of bacterial transformation and the presentation of new molecular biology techniques for this organism. We show that similarly to what has been demonstrated in other naturally competent bacteria, *Neisseria gonorrhoeae* can incorporate, at the same time, different DNA molecules modifying DNA at different loci within its genome. In particular, co-transformation of a DNA molecule bearing an antibiotic selection cassette and another non-selected DNA piece can lead to the integration of both molecules in the genome while selecting only through the selective cassette at percentages above 70%. We also show that successive selections with two selection markers at the same genetic locus can drastically reduce the number of genetic markers needed to do multisite genetic modifications in *Neisseria gonorrhoeae*. Despite public health interest heightened with the recent rise in antibiotic resistance, the causative agent of gonorrhea still does not possess a plethora of molecular techniques. This paper will extend the techniques available to the Neisseria community while providing some insights into the mechanisms behind bacterial transformation in *Neisseria gonorrhoeae*. We are providing a suite of new techniques to quickly obtain modifications of genes and genomes in the Neisserial naturally competent bacteria.

## 1. Introduction

*Neisseria gonorrhoeae* (Ng) is the causative agent of gonorrhea, the second most common human sexually transmitted disease, with worldwide yearly new cases of around 100 million (Kirkcaldy et al., 2019). As such, it has garnered intense scientific attention for decades. Yet the stakes have never been as high as now when a growing number of clinical isolates are resistant to antibiotics (Unemo and Shafer, 2014; Centers for Disease Control and Prevention (U.S.), 2019). The specter of a world with untreatable gonorrhea looms large (Ohnishi et al., 2011; Blomquist et al., 2014). Thus, the pace of study of *Neisseria gonorrhoeae* needs to speed up. One way to help is by providing quicker and more versatile molecular biology techniques for this genetically tractable organism. We will present here new means of performing genetic modifications in this important human pathogen that should be easily applicable to naturally competent members of the *Neisseria* genus.

Many bacteria like *Haemophilus influenzae*, *Vibrio cholerae* or *Streptococcus pneumoniae*, are naturally competent (Mell and Redfield, 2014). Yet the exact molecular mechanisms behind their ability to take up naked DNA from their environment are still murky despite close to a century of scientific inquiry. Frederick Griffith’s seminal work on transformation of *Streptococcus pneumoniae* was published in 1928 (Griffith, 1928). It wasn’t until 2014 that the bacterial machinery responsible for transformation was identified. This turned out to be a Type IV pilus (Tfp) (Laurenceau et al., 2013). Type IV pili are extremely dynamical polymers that can extend for micrometers away from the bacterial envelope (Berry and Pelicic, 2015; Craig et al., 2019). Tfp undergo cycles of extensions and retractions. Binding of a DNA molecule to the pilus and its subsequent retraction is supposed to be the first step of a series of coordinated molecular events culminating in the DNA molecule getting within the bacterial cytoplasm and ultimately integrated into the genome if enough homology exists (Hamilton and Dillard, 2006; Mell and Redfield, 2014; Piepenbrink, 2019). The terminology often distinguishes between pili and pseudopili mainly based on the extent of the extension of the appendage outside of the bacterial cell (Chen and Dubnau, 2004). Yet all these structures have a very close evolutionary relationship [now generally under the umbrella term type IV filaments (Berry and Pelicic, 2015; Piepenbrink, 2019)] and are a common feature of natural transformation.

*Neisseria gonorrhoeae* has emerged as a great model system for the study of Tfp. In *Neisseria gonorrhoeae*, Tfp are expressed throughout their life cycles and can represent as much as 100 micrometers of pili per cell (Biais et al., 2008). In *Neisseria gonorrhoeae*, Tfp are critical for many functions, beyond transformation: adhesion, motility and pathogenesis (Howie et al., 2005; Higashi et al., 2007; Taktikos et al., 2015; Hockenberry et al., 2016). Similarly to other naturally transformable bacteria, Tfp are paramount for *Neisseria gonorrhoeae* transformation. While for many bacteria, DNA will be transformed irrespective of its sequence, *Neisseria* species have short sequences referred to as DNA Uptake Sequences (Goodman and Scocca, 1988; Elkins et al., 1991) that can enhance uptake and are found in high abundance in their genomes (Treangen et al., 2008; Frye et al., 2013; Mora et al., 2021). The use of these sequences can enhance the transformation of desired DNA in the context of genetic engineering (Dillard, 2011; Duffin and Seifert, 2010). The exact nature of the interaction between the DNA molecule and the Tfp machinery is still the subject of active debate. The fact that a very small number of Tfp seems to maximize competence (Long et al., 1998) or the fact that pilins not assembled into polymers can still mediate transformation (Obergfell and Seifert, 2016) have, similarly to the case of pseudopilus, put into question the necessity for direct interaction between the DNA and the pilus. Whether direct or through the interaction of the minor pilin ComP (Berry and Cehovin, 2013; Cehovin et al., 2013; Berry et al., 2016), the specific interaction between DNA and Tfp is established (Hughes-Games et al., 2022). The study of the dynamics of intake of DNA molecules still argues for Tfp not being the sole structure to mediate DNA uptake in the periplasm (Hepp and Maier, 2016). Any new knowledge on the molecular mechanisms behind bacterial transformation will help optimize the transformation protocols and thus the efficiency of genetic manipulations in *Neisseria gonorrhoeae*.

One of the poorly characterized features of natural transformation is the amount of DNA that can be transferred within bacteria at the same time. Quantitation of fluorescently labeled DNA in *Neisseria gonorrhoeae* showed that one cell can harbor up to 40 kbp of DNA in its periplasm at once (Gangel et al., 2014). But the number of DNA molecules that can enter a given cell over the course of a transformation protocol is unknown. Can a cell acquire a second piece of DNA after acquiring a first one? With the prospect of easing genetic manipulation of both the *Vibrio* and *Streptococcus* genus, multiplex transformations have been performed with DNA molecules with homology with different loci across the genome (Dalia et al., 2014, 2017; Dalia, 2018). In the same spirit, we have decided to look at multi-molecule transformation in *Neisseria gonorrhoeae*.

In this article, we have first established different transformation protocols aimed at controlling the condition of contact between bacteria and the DNA molecules. In particular, we have designed a hybrid protocol stemming from existing techniques (Dillard, 2011) with attention to the concentration of DNA used and the conditions (liquid or agar plate) of the transformation. We have then quantified the co-transformation between DNA in two different genetic loci and show that co-transformation can occur at rates above 70%. We go on to present the logic behind an interchangeable cassette system where only two antibiotic selection markers are sufficient to make any number of successive genetic integrations throughout the *Neisseria gonorrhoeae* genome. We conclude by showing examples of the use of these techniques to obtain different mutants in *Neisseria gonorrhoeae*. This is our hope that these techniques will help speed up the much-needed unraveling of *Neisseria gonorrhoeae* general physiology in order to curb the prevalence of this important sexually transmitted disease while also presenting a framework for genetic manipulation in the *Neisseria* genus as a whole.

## 2. Materials and methods

### 2.1. Strains and growth conditions

#### 2.1.1. Growth conditions

The strains used in this study are all derivative of *Neisseria gonorrhoeae* strain MS11 (a gift from Magdalene So). All strains were grown on GCB agar plates supplemented with Supplements I and II at 37°C and 5% CO_2_. Nalidixic acid (3 μg/mL), kanamycin (80 μg/mL) or erythromycin (10 μg/mL) were supplemented to GCB agar plates when needed for selection.

#### 2.1.2. Construction of *GyrB1* MS11 mutant strain

The strain carrying the *gyrB1* mutation D429N (Duffin and Seifert, 2010) was obtained by PCR amplifying the MS11 *gyrB1* gene with the primers gyrB1_ampli_F et gyrB1_ampli_R (Supplementary Table S1) and cloned into a II-TOPO vector (Invitrogen). Site-directed mutagenesis (Quikchange, Stratagene) was performed on the plasmid to obtain the point mutation G to A leading to the D429N mutation with the primers gyrB1_MS11_F and gyrB1_MS11_R (Supplementary Table S1). This plasmid was transformed into the MS11 strain and selected to lead to the *GyrB1* MS11 mutant strain (MS11*_GyrB1_*).

#### 2.1.3. Construction of MS1 *AR::Kan*

The strain carrying the kanamycin resistance cassette was obtained by PCR amplifying a plasmid obtained from the Gene Synthesis services from Genscript Inc. found in Supplementary Table S2 with primers ResistanceF and ResistanceR (Supplementary Table S1). Roughly 400 bps on either side of the AR (Antibiotic Resistance) insertion point were PCR amplified with primer pairs UpGCF and UpGCR_resistance on one hand and DownGCF_resistance and DownGCR on the other hand. Fragments were joined by using Gibson Assembly Mix (New England Biolabs). The assembled product was further amplified by the external primers, transformed into MS11 and selected to obtain the *AR::Kan* MS11 strain (MS11*_AR::Kan_*).

#### 2.1.4. Construction of MS1 *AR::Erm*

To obtain MS11*_AR::Erm_* strain, Erythromycin cassette consists of Erythromycin resistance gene was amplified from MS11 Strain 306 (Trieu-Cuot et al., 1990) using AR_Erm_F and AR_Erm_R. Flanking regions around the AR insertion region was amplified as follows: the upstream region was amplified with UpGCF and UpGCR, the downstream region was amplified with DownGCF and DownGCR. These amplified fragments were then assembled using Gibson Assembly Mix (New England Biolabs). The assembled product was amplified through PCR using primers UpGCF and Down GCR. The product of this PCR was then transformed into MS11 and selected with erythromycin to yield the MS11*_AR::Erm_* strain.

### 2.2. Transformation assays

#### 2.2.1. Liquid transformation

Overnight cultures of *Neisseria gonorrhoeae* were obtained from streaking on GCB agar plates incubated at 37°C and 5% CO_2_. These cultures were resuspended in Transformation Medium (GCB + 5 mM MgSO_4_, Refer to Supplementary material for details). The cell suspension was used directly or diluted to 5 × 10^8^ CFU/mL. In this paper, extracellular DNA added for transformation will be called transformation DNA (tDNA). DUS tDNA was added to 200 μL of Transformation Medium in a separate tube and kept at 37°C. A volume of 30 μL of the cell suspension was then added to the DUS tDNA mixture. The combined product was incubated at 37°C for 15 min, in a 5% CO_2_ incubator. After incubation, the mixture was pipetted into a 60 mm × 15 mm petri dish containing 1 mL of pre-warmed GCB+ liquid medium (see Supplementary material for details). The Petri dishes were incubated at 37°C for 3 h, in a 5% CO_2_ incubator. After incubation, cells were scraped from the petri dish and resuspended in 1 mL of GCB liquid. The mixture was disrupted and prepared into serial dilutions. 5 μL spots of different dilutions were left to air dry on GCB and selection plates (refer to Supplementary material for details). The plates were then incubated at 37°C for 16–20 h, in a 5% CO_2_ incubator. Colonies were counted to measure transformation efficiency.

#### 2.2.2. Spot transformation

A known amount of DNA was prepared in a total volume of 10 μL and let to air dry on a GCB agar plate. A *Neisseria gonorrhoeae* colony was selected from an overnight culture and picked up with a swab. The swab was used to lawn bacteria uniformly across the marked spot where the DUS tDNA was air dried. The transformation plate was incubated at 37°C for 16–20 h, in a 5% CO_2_ incubator. All growth from the spot previously with dried DUS tDNA was swabbed and resuspended in 1 mL of GCB medium. The mixture was disrupted and prepared into serial dilutions. Dilutions were then spotted on GCB+ agar and selection plates and left to air dry. The plate was then incubated at 37°C overnight, in a 5% CO_2_ incubator. Colonies were counted to measure transformation efficiency.

#### 2.2.3. New spot transformation

An overnight culture of *Neisseria gonorrhoeae* on an agar plate was resuspended in GCB liquid medium adjusted to cell density with optical density at 600 nm wavelength (OD600) = 0.7 (approximately 5 × 10^8^ cells/ml). 10 μL of the culture was mixed with DUS tDNA. The mixture was then spotted on a GCB+ agar plate and left to air dry. Once the spot was air-dried, the agar plate was incubated at 37°C for 16 h, in a 5% CO_2_ incubator. After incubation, all growth from the spot was swabbed and resuspended in 1 mL of GCB medium. The mixture was disrupted and prepared into serial dilutions. Dilutions were then spotted on GCB and selection plates and left to air dry. The plate was then incubated at 37°C overnight, in a 5% CO_2_ incubator. Colonies were counted to measure transformation efficiency.

### 2.3. Optimizing OD600 for transformation

*Neisseria gonorrhoeae* bacteria grown overnight on GCB agar plate were swabbed and resuspended in 1 mL of GCB medium and disrupted to a single cell suspension using a disruptor for 2 min (Genie Cell Disruptor). A cell suspension with an OD600 of 0.7 was prepared from the stock. 10-fold serial dilutions were prepared from OD600 = 0.7 cell stock. 10 μL of each dilution was mixed with 500 ng of gyrB mutant DUS tDNA and transformed following the Spot and Dry transformation as illustrated in “New Spot Transformation.”

### 2.4. Co-transformation

This experiment used two types of tDNA: mutant *gyrB1* (gyrB1-2kbp, gyrB1-4kbp, and gyrB1-6kbp tDNA) and a Kanamycin-resistant cassette (AR::Kan tDNA). Mutant *gyrB* DNA were amplified from a Nalidixic acid resistance strain containing a point mutation within the *gyrB* gene (The details of the mutation will be illustrated in the Results section) (Duffin and Seifert, 2010), the MS11*_GyrB1_* strain. Different sizes of gyrB1 mutant tDNA were amplified to be 2kbp, 4kbp, and 6kbp using primers in Supplementary Table S1. The Kanamycin-resistant cassette was amplified from the strain MS11*_AR::Kan_* with primers Insert1kbGCF and Insert1kbGCR (Supplementary Table S1).

To compensate for the DNA copy number due to different molecule sizes, different amounts of tDNA were added depending on their sizes. In this set of experiments, all conditions used 0.1 ng of Kanamycin -resistant cassette. The amount of mutant *gyrB* tDNA used based on different sizes were listed in Supplementary Table S3.

### 2.5. Interchangeable cassette system

A specific region in the *Neisseria gonorrhoeae* genome has been selected for gene insertion based on previous studies (Dillard, 2011), between the lactate permease (*lctP*) gene and the aspartate aminotransferase (*aspC*) gene. This region was historically used as a complementation site and referred to later as the neisseria intergenic complementation site (nics) (Mehr and Seifert, 1998; Tobiason and Seifert, 2010). This region will be called the AR insertion region in this paper. Gene insertion in this region has shown no phenotypic changes in the bacteria (Ramsey et al., 2012). Cassettes containing selection markers are designed to have similar sized flanking regions on both sides of the AR insertion region. For transformation, the AR insertion region was amplified to ensure cassettes containing different selection markers have the same homologous recombination region. The cassettes (AR:: Kan or AR::Erm tDNA) used for study were amplified from strains containing respective selection markers (Supplementary Table S2) (either strain MS11*_AR::Kan_* or MS11*_AR::Erm_*) using primers listed in Supplementary Table S1. The transformation procedure used was the new spot transformation presented earlier.

### 2.6. Proof of concept for cloning using co-transformation

For examples intended to show deletion, we chose the *cidA* and *cidB* genes. We amplified the upstream DNA sequence around 3.2kbp and the downstream DNA sequence around 2kbp of *cidA/lrgA* and *lrgB* genes using the primers stated in Supplementary Table S1 (CidAB--153-F and CidAB-p73-R). After checking the PCR reaction on gel electrophoresis and clean-up, we stitched the two fragments together using Gibson Assembly mix. The assembled product was used as the template for PCR to get enough copies of the big fragments. The final PCR product was used directly with 1 ng of kanamycin AR cassette for co-transformation in the MS11 strain.

Next, to introduce a point mutation in the *pilD* gene, we did two sets of tests. For the first set, we amplified an upstream fragment and downstream fragment of the intended mutation (from CCTGCTGTCCCAAATGCCGTGTGCCG to CCTGCTGTCCC AAAAGCCGTGTGCCG representing a non-synonymous mutation pilDC72S), using primers stated in Supplementary Table S1. The fragments were then assembled using NEB HiFi DNA Assembly Master Mix. The assembled product was amplified again through PCR and the product was checked through gel electrophoresis. For the second set, we amplified a DNA fragment from mutant MS11 pilDC72S genomic DNA, which already carries the intended mutation, using Q5 High-Fidelity 2x Master Mix and primers stated in Supplementary Table S1. After ensuring good PCR results on gel electrophoresis, we co-transformed the fragment from both sets with 1 ng of erythromycin AR cassette into the MS11*_ΔcidAΔcidB AR::kan_* strain.

Following that, we intended to obtain a N-terminally tagged YFP-ComM protein. Similar to what we did for the point mutation, we introduced two sets of tests. The first set consisted of assembled DNA fragments, while the second set consisted of DNA fragments amplified directly from an existing mutant. For the first set, we located the *comM* gene in the MS11 strain. We amplified the YFP gene [gift from Lucy Shapiro (Viollier et al., 2004)] with the FluoComM-fluo-F and YFPComM-yfp-R including a linker. Two DNA fragment including the Upstream region of *comM* gene and the *comM* gene and part of its Downstream region were amplified, respectively, with the primer pairs ComM-Up-F and ComM-Up-R and ComM-Down-F and ComM-Down-R. The three fragments were assembled using NEB Hi-Fi DNA Assembly Kit. The product was further amplified by the primers ComM-Up-F and ComM-Down-R. PCR was performed using Q5 High-Fidelity 2x Master Mix with primers stated in Supplementary Table S1. For the second set, DNA fragments were amplified from MS11*_yfp-ComM AR::Erm_.* After checking for good amplified PCR product by gel electrophoresis, fragments from both sets were mixed with 1 ng of AR::Kan cassette and co-transformed into MS11*_ΔcidAcidB pilDC72S AR::Erm_* using conventional spot transformation. The transformed cells were lawned on an erythromycin selection agar plate. Colonies were selected the day after, resuspended in water, and used as the template for PCR using DreamTaq and colony PCR primers, ComM--525-F and ComM-787-R (refer to Supplementary Table S1). The PCR product result was loaded onto 1% agarose gel to check for colonies with successful insertion.

### 2.7. Statistical analysis and software used

*In-silico* work for genetic engineering was performed using software Geneious (Dotmatics) and VectorNTI 10 (Life Technologies). All visualization and statistical analysis were performed on Prism (Graphpad).

## 3. Results and discussion

### 3.1. Comparing different transformation assays

Being naturally competent, *Neisseria gonorrhoeae* bacterial cells do not need to be treated either chemically or physically to undergo transformation. The simple fact of putting into contact DNA molecules bearing homology with the genomes of *Neisseria gonorrhoeae* with *Neisseria gonorrhoeae* cells can allow the obtention of genetically modified bacteria. Yet, many factors can drastically affect the yield: the degree of homology, the specific strain used, the presence or absence of DNA Uptake sequences, the presence or absence of magnesium, the quantity of DNA and the concentration of cells used, to name a few parameters. But the exact methodology used to put into contact the DNA molecules and the bacterial cells also have an impact on the success of the transformation.

**Figure 1:**
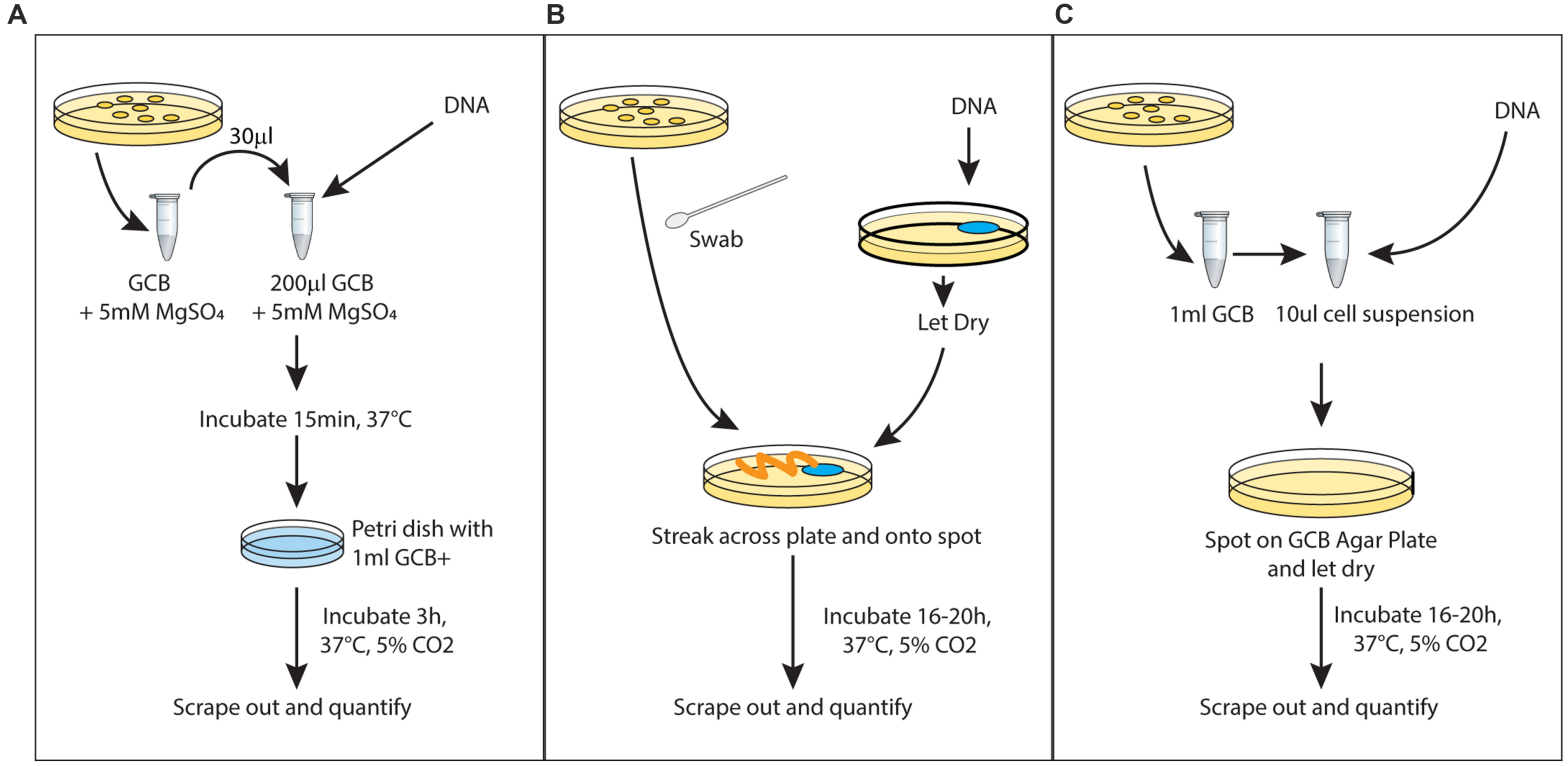
Different protocols for transformation: **(A)** liquid transformation, **(B)** spot transformation, and **(C)** dry with spot transformation.

Historically, there have been two commonly used lab techniques for transformation in *Neisseria gonorrhoeae*: Spot Transformation and Liquid Transformation (Dillard, 2011). Both methods are illustrated in Figure 1. In the Spot transformation, a known quantity of the DNA to be transformed (tDNA) is dried on an agar plate. Bacteria are overlaid on top of the dried tDNA spot using a swab. While this technique is versatile and requires minimal intervention from the experimenter, it has shortcomings when it comes to quantification of transformation efficiency. Indeed, it is difficult to ensure that all the bacteria that will be tested for transformation have been in contact with the same amount of DNA or even in contact at all with DNA. In the liquid transformation technique, a known quantity of tDNA is added to bacteria in liquid supplemented with magnesium. After a brief incubation, the DNA bacteria suspension is added to another volume of liquid and left in the incubator for a few hours. This technique ensures a uniform access between DNA and bacteria cells, but it requires many steps not easily scalable when many samples need to be handled at the same time. We have decided to combine these two techniques to allow an easy and accurate quantification of transformation. In a nutshell, we add tDNA to a suspension of bacteria cells of known density that is dried on an agar plate. In this case, all bacteria are thus put into contact with the tDNA. After some incubation time, the entirety of the DNA-bacteria spot is resuspended and transformation efficiency is assessed. This new methodology will be referred to as the new spot transformation.

**Figure 2:**
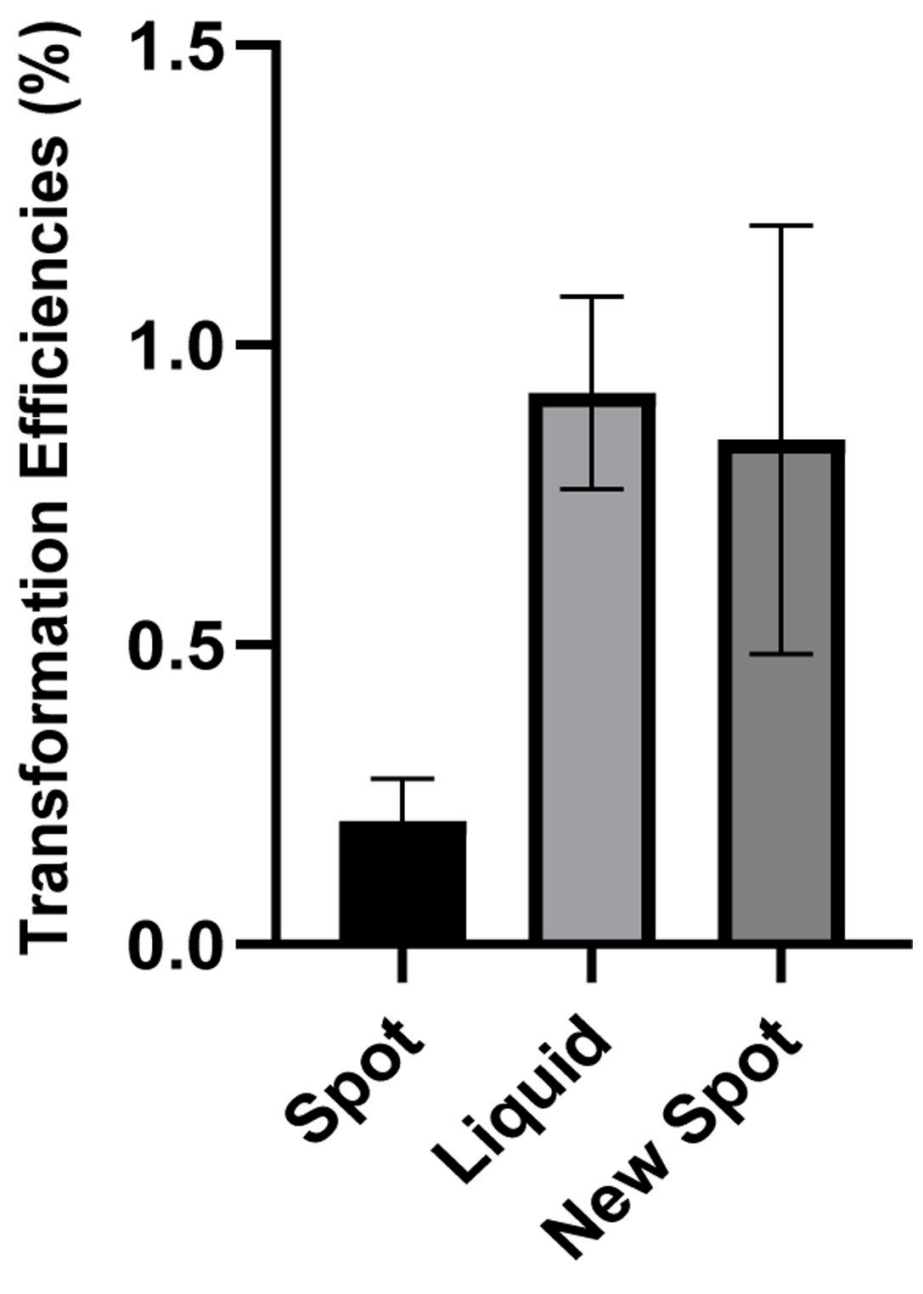
Comparison between three transformation protocols: Figure shows the transformation efficiency of three transformation protocol. Efficiency was calculated as (transformed bacteria number/total bacteria number)*100%. All experiments were performed in a minimum of three biological replicates with technical triplicates for each. The detection level of the transformation assays is below 5.10^−5^%. DNA free controls were below the detection limit.

In order to directly compare these different techniques, we have chosen one type of tDNA. The tDNA is a DNA carrying a point mutation in the Ng *gyrB* gene (gyrase B) known to confer resistance to the fluoroquinolone Nalidixic Acid (DUS tDNA). Throughout this series of experiments, 500 ng of mutant gyraseB (*gyrB*) DNA was used as transfer DNA (DUS tDNA). Since it is observed that more tDNA amount led to higher transformation, we standardized the amount due to its good yield, practicality of experiments and easy comparison across experiments. We can see in Figure 2 that the liquid transformation and the new technique can both achieve high transformation yield. The main advantage of the spot transformation technique is its technical ease, the limited steps involved and the possibility to process many samples in parallel. We see here that it does present a lower transformation yield. Thus, our new techniques represent a good middle ground between the two previously described techniques that maximizes the efficiency of transformation, minimizes the efforts of the experimenters and allows accurate quantification of transformation.

**Figure 3:**
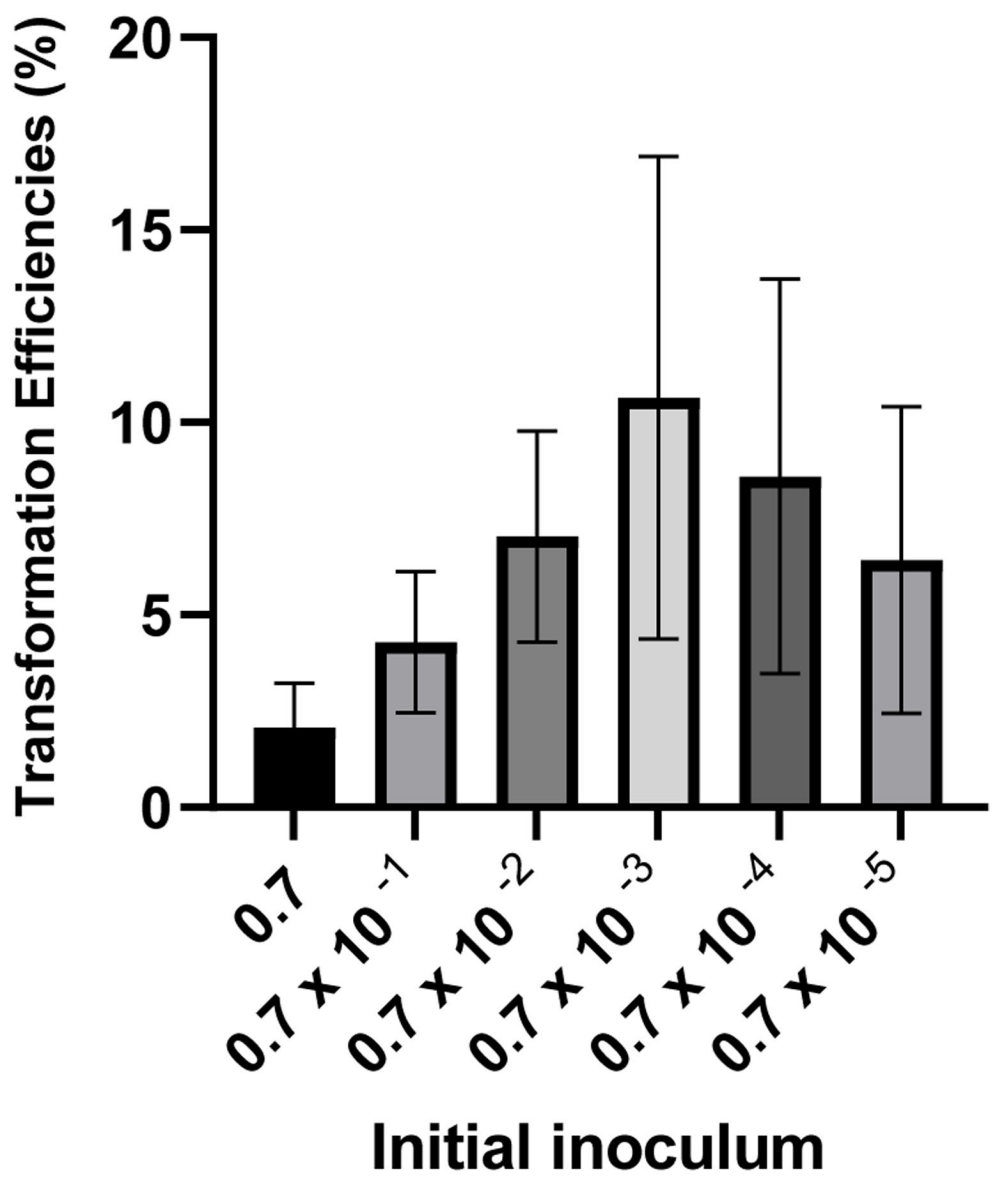
Transformation with different amounts of inoculant. Figure shows the fold change of transformation efficiencies of 10× dilutions of OD600 0.7 against OD600 0.7. Efficiency was calculated as (transformed bacteria number/total bacteria number)*100%. All experiments were performed in a minimum of three biological replicates with technical triplicates for each. The detection level of the transformation assays is below 5.10^−5^%. DNA free controls were below the detection limit.

There are several factors that can affect transformation efficiency in *Neisseria gonorrhoeae*. Their sheer number makes it difficult to tackle all of them at the same time. This includes the presence of competence pili, tDNA sizes, amount of DNA, and incubation time (Biswas et al., 1977). Some of these factors are also affecting transformation in much more heavily studied model systems like *E. coli* or *Bacillus subtilis.* Following the development of the new spot transformation aiming at quantification, we applied this newly established methodology to study the optimal cell number input for natural transformation fixing a few of those parameters. We have maintained the amount of tDNA (500 ng). The result shows that transformation using an input suspension of cells with an Optical Density of 0.0007 at 600 nm, together with 500 ng of tDNA yields a higher transformation efficiency as demonstrated in Figure 3. This suggests that there is an optimal cell to tDNA ratio in each transformation condition. In subsequent experiments in the present manuscript, this optimal input cell density is used.

### 3.2. Co-transformation

Compared to other genetically tractable bacterial organisms like *E. coli*, *Bacillus subtilis* or *Pseudomonas aeruginosa*, *Neisseria gonorrhoeae,* can be seen as possessing a limited array of genetic tools. While the natural competency status of *Neisseria gonorrhoeae* might have thwarted the efforts from the community to further optimize genetic modification techniques, this dearth of techniques might also be linked to mechanistic specificities of *Neisseria gonorrhoeae*. For instance, *Neisseria gonorrhoeae* does not have the plethora of cloning vectors designed and engineered for *E. coli*. To date, *Neisseria gonorrhoeae* can harbor three categories of plasmids, conjugative plasmids, β-lactamase plasmids and cryptic plasmids (Roberts, 1989; Cehovin et al., 2020). These plasmids are mainly known for their role in spreading antibiotic resistance among gonococci (Cehovin et al., 2020). In a laboratory setting, however, available vectors used for cloning in *Neisseria gonorrhoeae* research are largely limited to shuttle vectors for replication in *E. coli* (Ramsey et al., 2012). These vectors will eventually be cleaved upon uptake and reintegrated into its genome. They will have limited stability within Ng cells. Some of the early genetic manipulation of *Neisseria gonorrhoeae* rely largely on transforming the bacteria with genomic DNA, for example, acquiring streptomycin resistance after transforming with genomic DNA isolated from resistant strains (Sparling, 1966). As knowledge on DNA repair and recombination expands and with it an ever easier ability to create synthetic DNA molecules, studies moved toward transforming *Neisseria gonorrhoeae* with synthesized double-stranded DNA molecules containing the genomic manipulation of interest (insertion, deletion or point mutation), usually together with a selection marker with sometimes another step to remove it (Jones et al., 2022; Nyongesa et al., 2022). Yet, the selection schemes and the necessity for multiple antibiotic markers in the case of their use or the many steps when performing markerless modifications still impose many technical constraints on the genomic modification of *Neisseria gonorrhoeae* cells. We have decided to see if some of these constraints could be relaxed.

We have explored the possibility of multiplex genome editing in *Neisseria*, building on existing genetic manipulation protocols in other naturally competent organisms. Researchers succeeded in performing multiple-site genome editing at once in *Vibrio cholerae*, *V. natriegens* (Dalia et al., 2014, 2017). In a nutshell, multiple DNA molecules with homology with different parts of the genome of the targeted organisms are transformed simultaneously. Only one of the pieces will bear an antibiotic marker that will be selected for. Yet multiple mutations can be seen in the selected clones. *V. cholerae* and *N. gonorrhoeae* both perform natural transformation via type IV pili. We thus surmised that a similar experimental design will also work with *Neisseria gonorrhoeae* and has the potential to ease the design and execution of genomic modifications in this organism.

**Figure 4:**
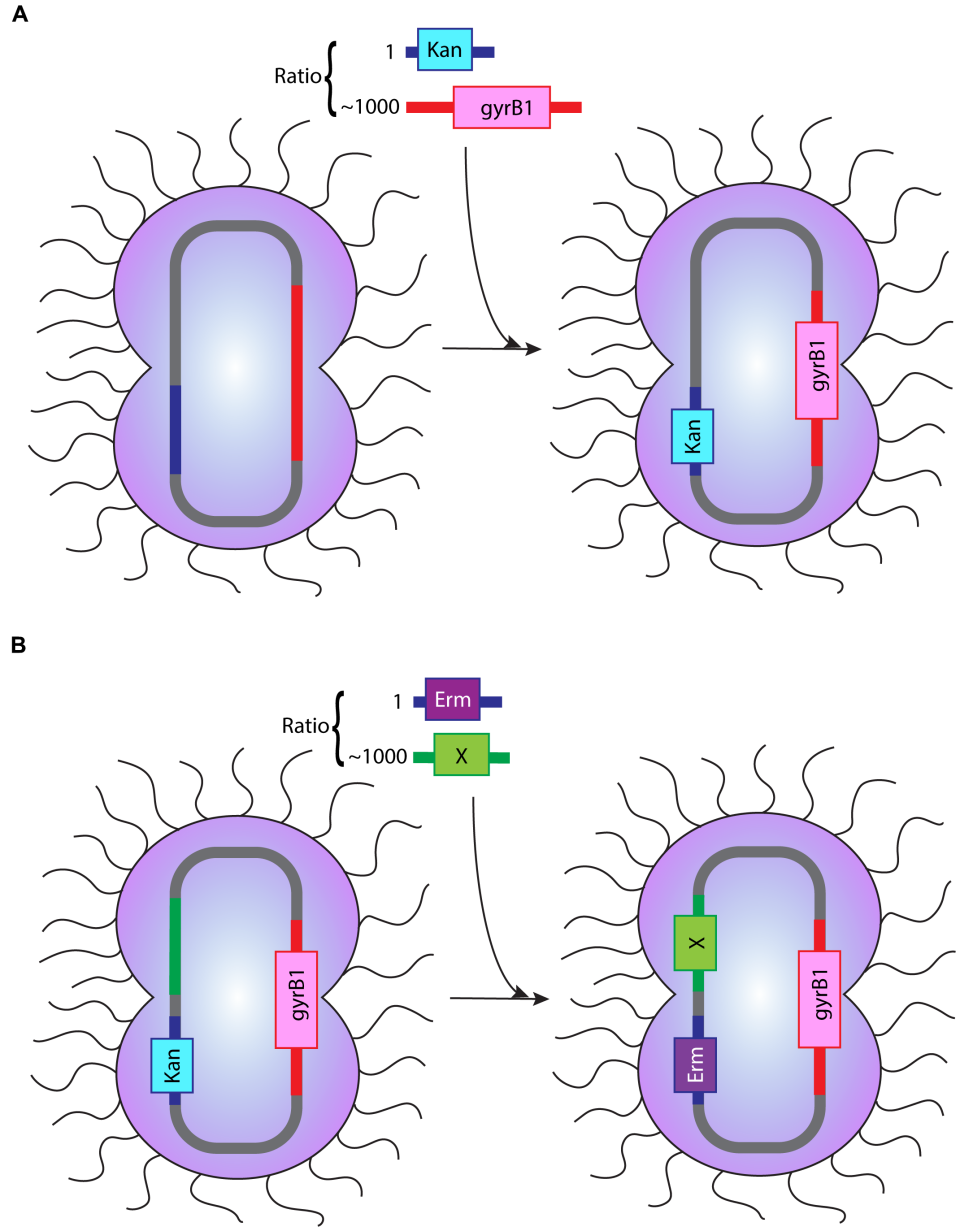
Depiction of the procedure of co-transformation **(A)** The procedure of co-transformation described in the “co-transformation” section. A tDNA with the Kan cassette:gyrB1 tDNA ratio of 1:1,000 was used to assess the effect of gyrB1 tDNA flanking region length in co-transformation. **(B)** The procedure of co-transformation when applied to cloning strategies. The image illustrated the application of Erm cassette:modification (labeled as X) with the ratio 1:1,000 into bacteria that has been co-transformed successfully prior. These two images depict a summary of co-transformation procedure and the possibility of subsequent co-transformation with a different selection cassette.

**Figure 5:**
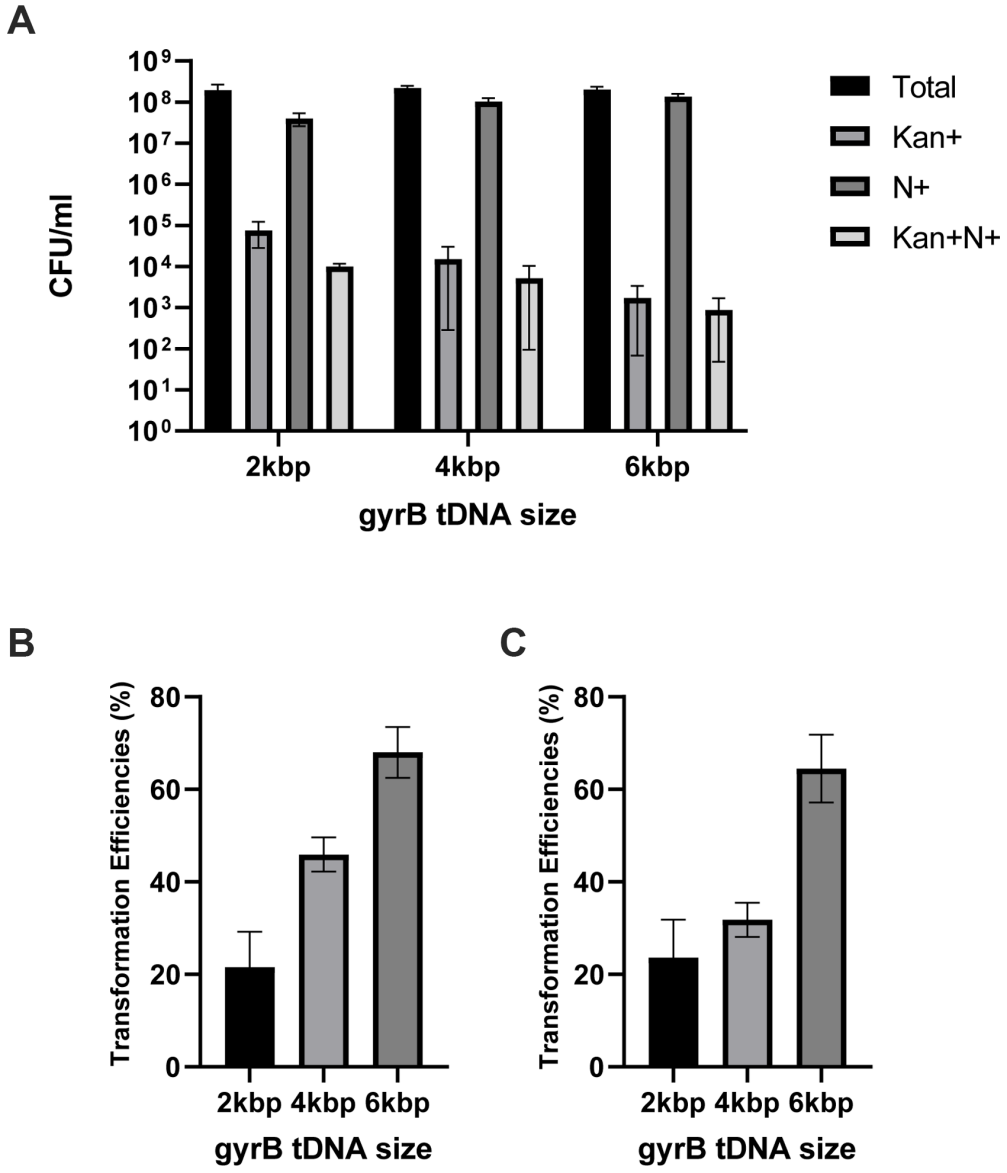
Transformation efficiencies using co-transformation **(A)** CFU/ml of cells transformed with tDNA of 2 kbp, 4 kbp, and 6 kbp length on different agar plate: GCB+ (Total), Kanamycin (Kan+), Nalidixic acid (N+) and Kanamycin and Nalidixic acid (Kan + N+) **(B)** Transformation efficiencies of nalidixic acid resistant cells over the total amount of cells for different tDNA length. Efficiency was calculated as (Nalidixic acid resistant cells/total bacteria number)*100%. **(C)** Percentage of Kanamycin and nalidixic acid resistant cells over Kanamycin resistant population in each co-transformation condition. Percentage was calculated as (Nalidixic acid resistant cells/Kanamycin resistant cells)*100%. All experiments were performed in a minimum of three biological replicates with technical triplicates for each. The detection level of the transformation assays is below 5.10^−5^%. DNA free controls were below the detection limit.

In order to test our hypothesis, we have chosen a site historically used to insert antibiotic markers in *Neisseria gonorrhoeae*, the region between the *lctP* and *aspC* genes (Ramsey et al., 2012), hereafter dubbed the AR (antibiotic resistance) region. We have paired this site with the *gyrB* point mutation presented in the previous section. We will combine these two types of tDNA in these co-transformation experiments mixing them together to perform a new spot transformation with optimized concentration of *Neisseria gonorrhoeae* cells. On the one hand, DNA molecules with various degrees of homology with the *lctP*-*aspC* gene region will allow the insertion of an antibiotic selection marker, the intended selection during co-transformation (AR::Kan tDNA). On the other hand, DNA molecules of various lengths carrying the point mutation conferring resistance to nalidixic acid (gyrB1-2kbp, gyrB1-4kbp, and gyrB1-6kbp) will allow us to measure how many cells selected with the AR gene also incorporated the gyrB point mutation. In this study, we used selection cassettes which include an antibiotic resistance (AR) gene, flanked with around 1,000 bp homologous regions on each side of the insertion site. Therefore, during successful insertion, the two homologous flanking regions will recombine with the genomic DNA and insert the antibiotic resistance gene at the desired location while conferring resistance to the chosen antibiotics, here kanamycin (Figure 4A). Selection of co-transformed colonies on kanamycin plates enables to measure the numbers of successful AR incorporation. Similarly, successful insertion at the *gyrB* region is measured by CFU on nalidixic acid selection plates and successful double mutants are measured by CFU on selection plates containing both the AR antibiotics (here kanamycin) and nalidixic acid. The ratio between these two different types of tDNA will be the main parameter in these experiments. The transformation efficiency of mutant gyrB tDNA increases as molecular size, and thus the degree of homology, increases (see Figure 5B). This observation is similar to what is reported in *V. natriegens* (Dalia et al., 2017). The increased pattern of transformation efficiency is most likely due to longer flanking regions available for recombination. It could also further enhance by a specificity of the *Neisseria* genus, the existence of DNA Uptake Sequences already mentioned. These 10–12 base pair sequences facilitate uptake and transformation of DNA molecules in the *Neisseria* genus (Goodman and Scocca, 1988; Elkins et al., 1991). DUS sequences occur on average every thousand base pairs in *Neisseria gonorrhoeae* and the number of their occurrences in a given tDNA molecule might also contribute to the increased transformation rate (Duffin and Seifert, 2010; Frye et al., 2013). With an experimental mindset, what is worth noting is the proportion of double mutants among the kanamycin resistant population. Even though we have only selected for kanamycin, as high as 60–70% of the cells are also resistant to nalidixic acid (see Figure 5C). Thus, if we were to perform co-transformation with an AR tDNA and another tDNA bearing a desired genomic modification that is not selectable, we can still obtain the incorporation of that not selectable genomic mutation by solely selecting with the AR antibiotic. This scheme opens up fully the design of genetic modifications, as we will see later, while providing a method requiring only one transformation step.

This observation is of extreme practical use since it provides an opportunity for new flexibility when manipulating the *Neisseria gonorrhoeae* genome. During random bacterial genetic manipulation, it is quite rare that the genetic change thought after will lead to any selectable or recognizable phenotype. The goal is more often to remove a gene and look for the potential phenotype associated with the removal in order to ascribe a function to the gene. Thanks to the high rate of co-transformation seen above, we see that it is technically possible to always use the same genetic locus to receive a selectable marker and get a high rate of modification in a totally independent locus on the genus that you are not selecting for. This opens the door for a quick and easy methodology for obtaining genome-wide modifications in *Neisseria gonorrhoeae*.

### 3.3. Interchangeable cassette system

**Figure 6:**
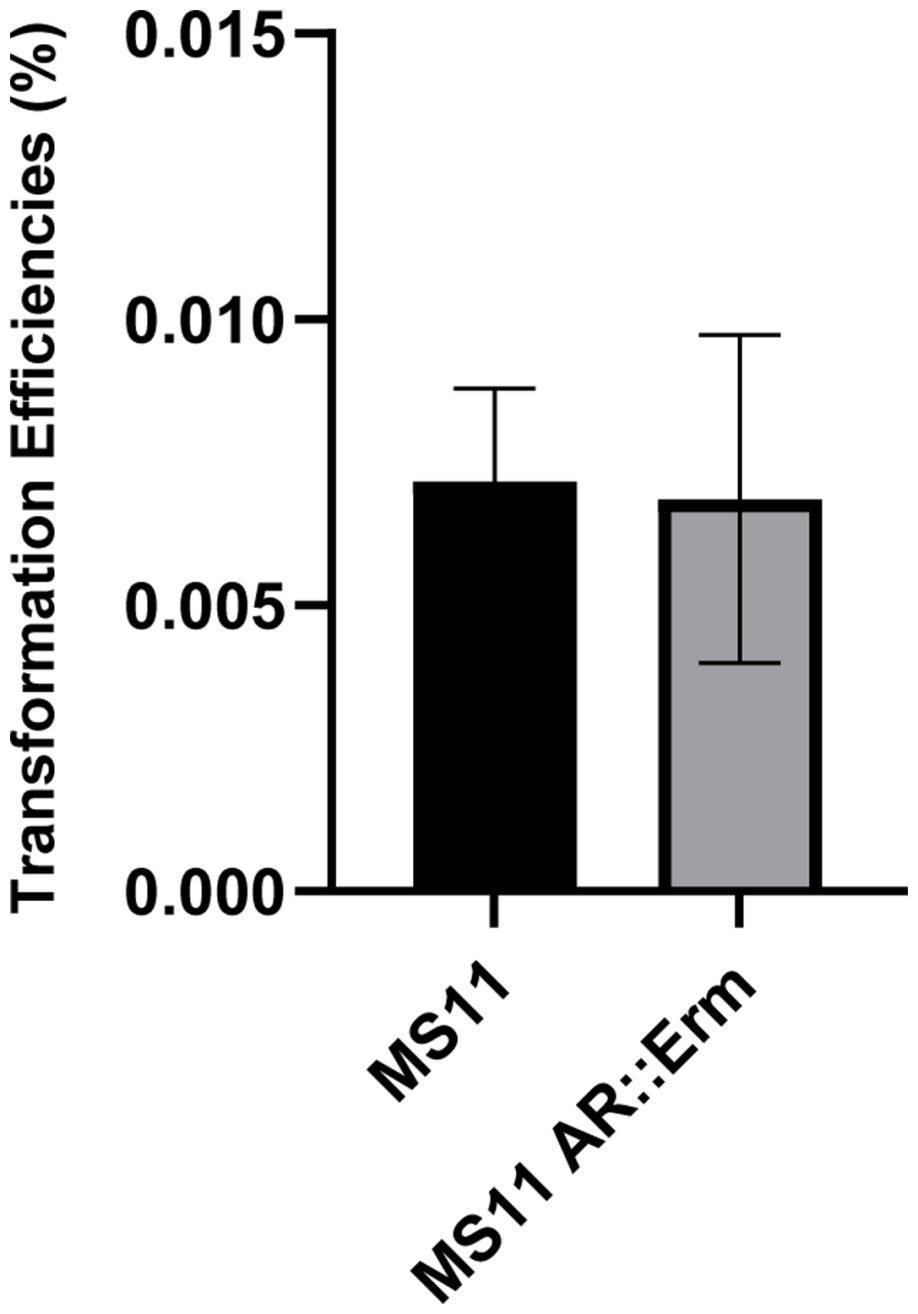
Consistency of AR region recombination Figure shows the transformation efficiency of transforming MS11 and MS11*_AR::Erm_* with 1 ng of AR cassette containing Kanamycin selection. Transformation efficiency was calculated as (Kanamycin resistant cells/total bacteria number)*100%.

Following the results on co-transformation from the previous section, the possibility of performing multiplex genome editing opens up. One of the advantages stemming from a co-transformation technique is the use of only one selection marker to obtain multiple-site manipulation. Given the limited number of selection markers that are available in *Neisseria*, we wondered whether we could use co-transformation to limit the number of selection markers used without limiting the number of mutations we could obtain. One appealing method would be to keep using the same historical locus to sequentially integrate different selection markers. We thus investigated the versatility of the AR region by switching in cassettes with different selection markers. We compared the transformation of kanamycin selection cassette (AR::Kan tDNA) into wild type (MS11) and a strain that possessed an erythromycin resistance cassette at the AR locus (MS11*_AR::Erm_*). Our results showed that the transformation efficiency between the two are similar (Figure 6). This result shows that an AR region inserted with a selection cassette is equally likely to recombine with a new selection cassette when given the opportunity. This finding implies the possibility to indeed apply co-transformation for multiple sequential genetic manipulations. Besides, it is possible to perform limitless sequential co-transformations with as little as two types of selection cassettes by using them alternately. For instance, we can limit ourselves to just the kanamycin cassette and erythromycin cassettes presented here and use the one not present at the AR locus to select for the next co-transformation, leading to the desired genetic modification(s) at any other site in the genome. Furthermore, we have verified that the insertion of the different cassettes in the AR region happens with the same frequency (see Figure 6). We hope that the use of this Interchangeable Cassette System (ICS) will prove useful in the *Neisseria gonorrhoeae* research arena as a means to reduce the use of antibiotic resistance makers and provide an efficient technique to obtain multisite modifications with limited steps and maximized efficiency.

### 3.4. Proofs of concept and rules of thumb: use of co-transformation for genetic manipulations

**Figure 7:**
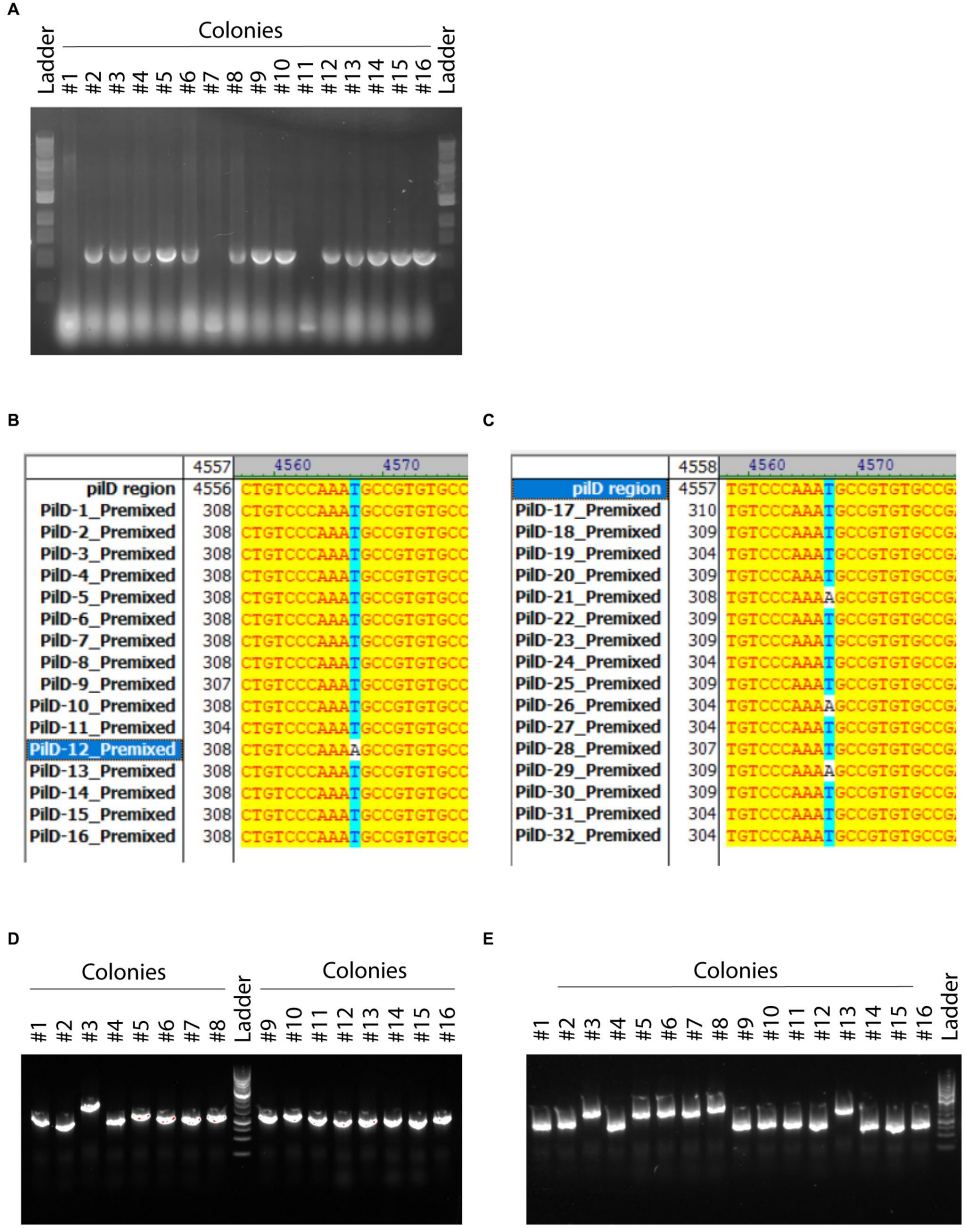
Application of co-transformation in creating mutants by different means **(A)** Deletion. The genes *cidA* and *cidB* were selected to be deleted. Transform DNA flanking upstream of *cidA* and downstream of *cidB* was assembled and transformed into MS11 strain. Agarose gel shows the product of colony PCR. Successful mutants could be observed with smaller molecular size. The DNA marker used in this image is the Quick-Load Purple 1 kb DNA Ladder (New England Biolabs). **(B)** Point mutation: co-transformation of amplified assembled DNA fragments. The figure shows the sequencing results compared to the original PilD sequence. A successful mutation was marked by the change from AAATGCCGTG to AAAAGCCGTG. **(C)** Point mutation: co-transformation of direct amplification product from genomic DNA. The figure shows the sequencing results compared to the original PilD sequence. A successful mutation was marked by the change from AAATGCCGTG to AAAAGCCGTG. **(D)** Insertion: co-transformation of amplified assembled DNA fragments. Each lane shows colony PCR of one single colony of transformants. A successful insertion was marked by the bigger DNA size on agarose gel. **(E)** Insertion: co-transformation of direct amplification product from genomic DNA. Each lane shows colony PCR of one single colony of transformants. A successful insertion was marked by the bigger DNA size on agarose gel. The DNA marker used in **(D,E)** is the GeneRuler 1 kb DNA Ladder (ThermoScientific).

In order to show the versatility of these techniques, we will illustrate it in common experimental situations. Here, we present three common types of genetic editing: (1) deletion, (2) point mutation, and (3) insertion. Besides the type of modifications, the examples are also coupled with tDNA prepared from different sources, namely amplified DNA fragments from DNA assembly or genomic DNA to further exemplify the flexibility of the methods to respond to the reality of a wide range of molecular biology applications. On top of that, this proof of concept illustrated the versatility of this gene modification workflow by performing all three types of gene editing consecutively through co-transformation. For the first example, we demonstrated gene deletion by co-transformation. We amplified two PCR fragments, one 3.2kbp upstream of the *cidA* gene and another 2 kbp downstream of the *cidB* gene, which were fused and amplified. We obtained a PCR product with the desired deletion of both *cidA* and *cidB*. After co-transformation with AR::Kan cassette into MS11 strain, 16 colonies were selected for colony PCR and checking. The amplified region spans across 153 bp upstream of the start codon of the *cidA* gene and 73 bp downstream of the stop codon of the *cidB* gene. Therefore, the expected band will be 1,263 bp (smaller band near the bottom) without the deletion and 226 bp when the deletion occurs. The deletion was further confirmed by sequencing. We observed 3 out of 16 colonies selected through the co-transformed selection (kanamycin) also carry the deletion of *cidA* and *cidB* (Figure 7A). By doing this first genomic manipulation, we obtained MS11*_ΔcidAcidB AR::Kan_*. We followed up the procedure by introducing a point mutation in a gene named *pilD*. The mutation intended was from CCTGCTGTCCCAAATGCCGTGTGCCG to CCTGCTGTCCCA AAAGCCGTGTGCCG leading to the non-synonymous pilDC72S mutation. We tested two forms of co-transformation: amplified products from DNA assembly and genomic DNA carrying the mutation. These arrangements will reflect well the two most common sources of implementing genetic modifications: introducing mutations by assembling DNA fragments or amplifying DNA fragments from another strain that already carry the intended mutation. After obtaining the intended tDNA with the point mutation of T- > A, we co-transformed these fragments with AR::Erm cassette into MS11*_ΔcidAcidB AR::Kan_*. Due to the nature of point mutations, successful mutations could not be differentiated by colony PCR and gel electrophoresis alone. To check the success rate of co-transformation, the amplified products of colony PCR were cleaned up and sent for sequencing. The success rate for the assembled DNA set was 1 out of 16 (Figure 7B), and 3 out of 16 for the direct amplification set. From this experiment, we obtained MS11*_ΔcidAcidB pilDC72S AR::Erm_*. At the same time, we also proved the possibility of switching the selection cassette, a great application of the rationale in designing the Interchangeable Cassette System (ICS). As an example of the last common genomic modification, we completed this section with a gene insertion. In this example, we also tested two forms of co-transformation as previously. We prepared tDNA amplified either from assembled DNA or genomic DNA carrying the intended modification. From that, the PCR product was co-transformed with AR::Kan cassette into MS11*_ΔcidAcidB pilDC72S AR::Erm_*. After selecting 16 colonies from each set, the success rate of co-transformation was checked through gel electrophoresis. The expected band for unsuccessful insertion and successful insertion are 1,312 bp and 2032 bp, respectively. We obtained 1 successful mutant out of 16 colonies for those from assembled product (Figure 7D) and 6 successful mutants out of 16 colonies for those from direct amplification (Figure 7E). Therefore, the successful mutants are now MS11*_ΔcidAcidB pilDC72S yfp-ComM AR::Kan_*.

Through these few examples, we showed the flexibility of co-transformation using DNA molecules from different sources, different homology lengths, for different purposes. While the success rate varies greatly with the specifics of the genetic manipulation at hand, the success rate is compatible with a rapid obtention of the desired mutation. A more systematic study of co-transformation, beyond the scope of the present work, might help to further unravel the reasons for these discrepancies. It could be that the recombination rates across the genome are not uniform, the stability of the DNA molecules getting inside the cell might depend on their sequence, the uptake of all the DNA molecules might not be equal to their relative concentration outside the cell, as a few possible contributors to the differences. But a few key guiding principles have ensured successful co-transformation: maximize the ratio of the non-selected tDNA to the selected one, have more homology in the non-selected piece than the selected one and minimize the amount of selected tDNA aiming for a few hundred selected clones per co-transformation. Moreover, the utilization of ICS in this proof of concept also proves the possibility of performing consecutive genetic modification with as little as two types of selection cassette. Plus, this system is compatible with co-transformation throughout three different genetic modifications. With these rules of thumb in mind, co-transformation is a highly flexible and rapid tool for genetic engineering in *Neisseria gonorrhoeae*, minimizing the design and steps required by other techniques.

## 4. Conclusion

There is a renewed interest in the study of *Neisseria gonorrhoeae* due to its rising antibiotic resistance. It is thus important to have efficient and rapid molecular biology tools in order to push forward the study of this important human pathogen. Comparatively to other bacterial model systems, Neisseriaceae lag behind in terms of diversity of genetic manipulation tools available. The rekindled interests both in terms of human pathogens or as emerging models for bacterial physiology have pushed for an expansion of the repertoire of genetic tools in Neisseria species. Animated by the same goal and inspired by the work of co-transformation of multiple DNA molecules in *Vibrio* species, we have decided to contribute to the further widening of genetic manipulation methodologies of *Neisseria gonorrhoeae*.

In the present study, we presented a modified transformation protocol aimed at maximizing transformation efficiency with minimal experimental steps involved while maintaining the freedom to perform any scar-free genetic modifications in *Neisseria gonorrhoeae*. We used this protocol to test the possibility to obtain multisite transformations when using multiple DNA molecules simultaneously. And indeed, *Neisseria gonorrhoeae* cells were able to integrate DNA from different molecules leading to a robust, rapid and flexible methodology to modify the *Neisseria gonorrhoeae* genome (see Figure 4A). Co-transformation with a set of only two interchangeable antibiotic cassettes provides a new means to engineer *Neisseria gonorrhoeae* species endlessly genetically (see Figure 4B). As the methodology is mostly dependent on the ability of *Neisseria gonorrhoeae* to be naturally competent, we have no doubt that this methodology will readily apply to other competent Neisseriaceae while contributing to the need for more genetic tools in *Neisseria gonorrhoeae*.

## Data availability statement

The original contributions presented in the study are included in the article/Supplementary material, further inquiries can be directed to the corresponding author.

## Author contributions

VS and NB designed and planned the research and wrote the manuscript. VS, NB, and OT performed and analyzed experiments. All authors contributed to the article and approved the submitted version.

## Funding

NB acknowledges funding from NIH grant AI116566 alongside a grant from the I-Bio Initiative at Sorbonne Université.

## Conflict of interest

The authors declare that the research was conducted in the absence of any commercial or financial relationships that could be construed as a potential conflict of interest.

## Publisher’s note

All claims expressed in this article are solely those of the authors and do not necessarily represent those of their affiliated organizations, or those of the publisher, the editors and the reviewers. Any product that may be evaluated in this article, or claim that may be made by its manufacturer, is not guaranteed or endorsed by the publisher.
