# Supplementary material for: Multisite transformation in Neisseria gonorrhoeae: insights on transformations mechanisms and new genetic modification protocols
Source: Front Microbiol. 2023 Jun 20;14:1178128. doi: 10.3389/fmicb.2023.1178128 (PMC10319059; doi:10.3389/fmicb.2023.1178128)

Supplementary Material

Multisite Transformation in *Neisseria gonorrhoeae:* insights on transformations mechanisms and new genetic modification protocols

Nicolas Biais*, Vui Yin Seow, Olga Tsygelnytska

*** Correspondence:** Nicolas Biais: nicolas@mechano-micro-biology.org

# Supplementary Tables

Table S1: Oligos used in this study

| **Oligo Name** | **Oligo Sequence** | **Remarks** |
| --- | --- | --- |
| gyrB1_MS11_F | CCTCGTCGAGGGCAACTCCGCAGGCGG | *gyrB1* mutation |
| gyrB1_MS11_R | CCGCCTGCGGAGTTGCCCTCGACGAGG | *gyrB1* mutation |
| GyrB1DUS | TCGTATGCCGTCTGAACCGCCGAAGTCATCATGAC | tDNA preparation |
| GyrB1Reverse | GCATTTGGCGGTAGAAGAAG | tDNA preparation |
| ResistanceF | CTCGAGGGCTTGACACTTTATG | Kanamycin cassette |
| ResistanceR | ATCGATGTTTAAACTTCAGACGGC | Kanamycin cassette |
| UpGCF | CCCCGCCAAACAAATGCCG | Kanamycin cassette |
| UpGCR_resistance | CATAAAGTGTCAAGCCCTCGAGGGGTATAGAGCAGAACGGATG | Kanamycin cassette |
| DownGCF_resistance | TCTGAAGTTTAAACATCGATCGTTTCCGCCTACCTCGAAC | Kanamycin cassette |
| DownGCR | CTGGAAACAGACGCAATCACG | Kanamycin cassette |
| AR_Erm_F | CCGTTCTGCTCTATACCCCTTAGAAGCAAACTTAAGAGTG | Erythromycin cassette |
| AR_Erm_R | GTTCGAGGTAGGCGGAAACGATCGATACAAATTCCCCG | Erythromycin cassette |
| UpGCF | CCCCGCCAAACAAATGCCG | Erythromycin cassette |
| UpGCR | GGGTATAGAGCAGAACGGATG | Erythromycin cassette |
| DownGCF | CGTTTCCGCCTACCTCGAAC | Erythromycin cassette |
| DownGCR | CTGGAAACAGACGCAATCACG | Erythromycin cassette |
| gyrB_2kbp_F | TACAAAATCTCCGGCGGCCTGCAC | Co-Transformation |
| gyrB_2kbp_R | GGTTCGACCTCGTCGCCCATCA | Co-Transformation |
| gyrB_4kbp_F | AGTTCTTTTGTCGCCACGACGAC | Co-Transformation |
| gyrB_4kbp_R | CCTGCGGTGAGCAGGAAGTTTTC | Co-Transformation |
| gyrB_6kbp_F | CAAGCGGTGCGGCATCGTCAAA | Co-Transformation |
| gyrB_6kbp_R | TGCCTCGCCTTAGCTCAAAGAGAA | Co-Transformation |
| ReadInsertGC_F | CGGCAATGATTTTCTTCCTC | Co-Transformation |
| ReadInsertGC_R | GGCTTCGATGTGCTTGAAGA | Co-Transformation |
| Insert1kbGCF | ATGGGTGCGCTGATTATGGT | ICS |
| Insert1kbGCR | AATCATGCCGTCCCAATCCA | ICS |
| ComM-Up-F | CGTAAGACACCAGTCCCAATAC | Co-Transformation Application: YFP-ComM |
| ComM-Up-R | ATTTCAGACGGCCTTATTCGC | Co-Transformation Application: YFP-ComM |
| FluoComM-fluo-F | GCGAATAAGGCCGTCTGAAATATGGTGAGCAAGGGCG | Co-Transformation Application: YFP-ComM |
| YFPComM-yfp-R | TCCACCTGCTCCACCTGCTCTAGACTTGTACAGCTCGTCC | Co-Transformation Application: YFP-ComM |
| ComM-Down-F | GCAGGTGGAGCAGGTGGATCGCTTGCCTTGGTTTACAG | Co-Transformation Application: YFP-ComM |
| ComM-Down-R | GACCGCAGCAGCCATTCTTG | Co-Transformation Application: YFP-ComM |
| ComM--525-F | CTTGTAATGTAATCGGGCGTTGC | Colony PCR primer for YFP-ComM |
| ComM-787-R | CGAGTTGTTGTTGGTGGTTGG | Colony PCR primer for YFP-ComM |
| deltaCidAB-Up-F | GCGAAGTGGTGCTGCGTTATCTG | Co-Transformation Application: CidA/B |
| deltaCidAB-Up-R | GTTGTGTGTCTGTCTTTTGACGGCGTTG | Co-Transformation Application: CidA/B |
| deltaCidAB-Down-F | GTCAAAAGACAGACACACAACACCCGTTTCAGACGGC | Co-Transformation Application: CidA/B |
| deltaCidAB-Down-R | CCGCCGTTGAAACCCGAAAAATAC | Co-Transformation Application: CidA/B |
| CidAB--153-F | AAAGTCTGAACACGCCCCGG | Co-Transformation Application: CidA/B Colony PCR |
| CidAB-p73-R | CAGCCATAACGGTTCTCCTTGCG | Co-Transformation Application: CidA/B Colony PCR |
| GCPilDC72S-Up-F | GGCAATCAGTGCCATCAGAACGGG | Co-Transformation Application: PilD |
| GCPilDC72S-Down-R | ATTCACCGTCCTTACGGGATGGTGC | Co-Transformation Application: PilD |
| GCPilDUpNewF | CGGTTCGCTTGGTGCTTCATC | Co-Transformation Application: PilD |
| GCPilDDownNewR | TGGTCAGGACACGCCGTATCAG | Co-Transformation Application: PilD |
| GCPilDC72S-Up-NR | GCGCGTATCGGCACACGGCTTTTGGGACAGCAG | Co-Transformation Application: PilD |
| GCPilDC72S-Down-NF | CCTGCTGTCCCAAAAGCCGTGTGCCGATAC | Co-Transformation Application: PilD |

Table S2: Gene Sequence

| **Gene Name** | **Gene Sequence** | **Source** |
| --- | --- | --- |
| Kanamycin resistance cassette | CTCGAGGGCTTGACACTTTATGCTTCCGGCTCGTATAATGTGTGGATAGTGGGAGGAAAGCATGATTGAACAAGATGGATTGCACGCAGGTTCTCCGGCCGCTTGGGTGGAGAGGCTATTCGGCTATGACTGGGCACAACAGACAATCGGCTGCTCTGATGCCGCCGTGTTCCGGCTGTCAGCGCAGGGGCGCCCGGTTCTTTTTGTCAAGACCGACCTGTCCGGTGCCCTGAATGAACTGCAGGACGAGGCAGCGCGGCTATCGTGGCTGGCCACGACGGGCGTTCCTTGCGCAGCTGTGCTCGACGTTGTCACTGAAGCGGGAAGGGACTGGCTGCTATTGGGCGAAGTGCCGGGGCAGGATCTCCTGTCATCCCACCTTGCTCCTGCCGAGAAAGTATCCATCATGGCTGATGCAATGCGGCGGCTGCATACGCTTGATCCGGCTACCTGCCCATTCGACCACCAAGCGAAACATCGCATCGAGCGAGCACGTACTCGGATGGAAGCCGGTCTTGTCGATCAGGATGATCTGGACGAAGAGCATCAGGGGCTCGCGCCAGCCGAACTGTTCGCCAGGCTCAAGGCGCGCATGCCCGACGGCGAGGATCTCGTCGTGACCCATGGCGATGCCTGCTTGCCGAATATCATGGTGGAAAATGGCCGCTTTTCTGGATTCATCGACTGTGGCCGGCTGGGTGTGGCGGACCGCTATCAGGACATAGCGTTGGCTACCCGTGATATTGCTGAAGAGCTTGGCGGCGAATGGGCTGACCGCTTCCTCGTGCTTTACGGTATCGCCGCTCCCGATTCGCAGCGCATCGCCTTCTATCGCCTTCTTGACGAGTTCTTCTGAGCCGTCTGAAGTTTAAACATCGAT | Genescript Plasmid |
| Erythromycin Resistance Cassette | CTTAGAAGCAAACTTAAGAGTGTGTTGATAGTGCAGTATCTTAAAATTTTGTGTATAATAGGAATTGAAGTTAAATTAGATGCTAAAAATTTGTAATTAAGAAGGAGGGATTCGTCATGTTGGTATTCCAAATGCGTAATGTAGATAAAACATCTACTGTTTTGAAACAGACTAAAAACAGTGATTACGCAGATAAATAAATACGTTAGATTAATTCCTACCAGTGACTAATCTTATGACTTTTTAAACAGATAACTAAAATTACAAACAAATCGTTTAACTTCTGTATTTATTTACAGATGTAATCACTTCAGGAGTAATTACATGAACAAAAATATAAAATATTCTCAAAACTTTTTAACGAGTGAAAAAGTACTCAACCAAATAATAAAACAATTGAATTTAAAAGAAACCGATACCGTTTACGAAATTGGAACAGGTAAAGGGCATTTAACGACGAAACTGGCTAAAATAAGTAAACAGGTAACGTCTATTGAATTAGACAGTCATCTATTCAACTTATCGTCAGAAAAATTAAAACTGAACATTCGTGTCACTTTAATTCACCAAGATATTCTACAGTTTCAATTCCCTAACAAACAGAGGTATAAAATTGTTGGGAGTATTCCTTACCATTTAAGCACACAAATTATTAAAAAAGTGGTTTTTGAAAGCCATGCGTCTGACATCTATCTGATTGTTGAAGAAGGATTCTACAAGCGTACCTTGGATATTCACCGAACACTAGGGTTGCTCTTGCACACTCAAGTCTCGATTCAGCAATTGCTTAAGCTGCCAGCGGAATGCTTTCATCCTAAACCAAAAGTAAACAGTGTCTTAATAAAACTTACCCGCCATACCACAGATGTTCCAGATAAATATTGGAAGCTATATACGTACTTTGTTTCAAAATGGGTCAATCGAGAATATCGTCAACTGTTTACTAAAAATCAGTTTCATCAAGCAATGAAACACGCCAAAGTAAACAATTTAAGTACCATTACTTATGAGCAAGTATTGTCTATTTTTAATAGTTATCTATTATTTAACGGGAGGAAATAATTCTATGAGTCGCTTTTTTAAATTTGGAAAGTTACACGTTACTAAAGGGAATGGAGATAAATTATTAGATATACTACTGACAGCTTCCAAGAAGCTAAAGAGGTCCCTAGCGCCTACGGGGAATTTGTATCGAT | MS11 Strain 306 |

Table S3: Amount of mutant gyrB tDNA based on different tDNA sizes

| **gyrB DNA size** | **gyrB DNA (ng) used** |
| --- | --- |
| 2kbp | 100 |
| 4kbp | 200 |
| 6kbp | 300 |

Table S4: tDNA used in this study

| **tDNA** | **Description** |
| --- | --- |
| DUSgyrB1 | Amplified from MS11 *gyrB1* mutant using GyrB1DUS and GyrB1Reverse |
| gyrB1-2kbp | Amplified from MS11 *gyrB1* mutant using gyrB_2kbp_F and gyrB_2kbp_R |
| gyrB1-4kbp | Amplified from MS11 *gyrB1* mutant using gyrB_4kbp_F and gyrB_6kbp_R |
| gyrB1-6kbp | Amplified from MS11 *gyrB1* mutant using gyrB_6kbp_F and gyrB_6kbp_R |
| AR::Kan | Amplified from MS11*_AR::Kan_* using Insert1kbGCF and Insert1kbGCR |
| AR::Erm | Amplified from MS11*_AR::Erm_* using Insert1kbGCF and Insert1kbGCR |

# Supplementary Figures

Supplementary Figure S1: Positions and numbers of DUS sequences in the GyrB1 tDNAs


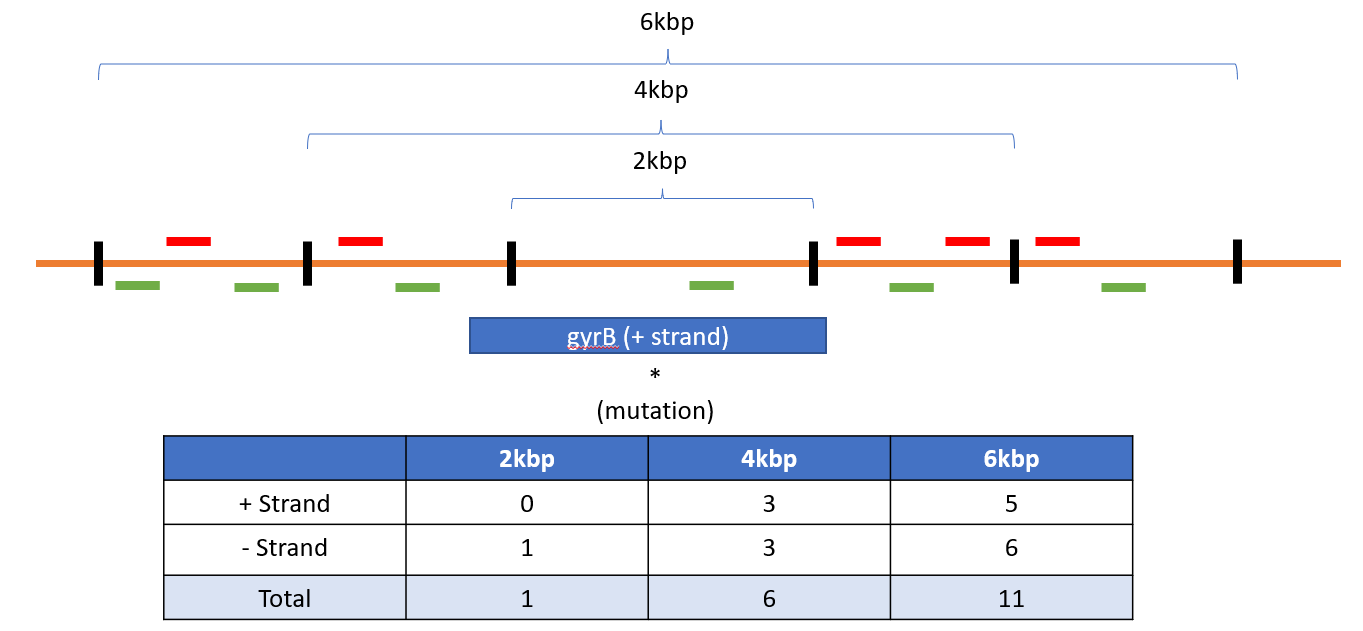

Supplement: Supplementary file 1 [file Data_Sheet_1.docx]
